# Expression Pattern of p62 in Primary Age-Related Tauopathy: Staging of p62 in PART

**DOI:** 10.3389/fnagi.2022.793353

**Published:** 2022-04-25

**Authors:** Xin Wang, Lei Zhang, Hui Lu, Juanli Wu, Huazheng Liang, Bing Sun, Keqing Zhu

**Affiliations:** ^1^China Brain Bank and Department of Neurology in Second Affiliated Hospital, Key Laboratory of Medical Neurobiology of Zhejiang Province, and Department of Neurobiology, Zhejiang University School of Medicine, Hangzhou, China; ^2^Department of Pathology, Zhejiang University School of Medicine, Hangzhou, China; ^3^Translational Research Institute of Brain and Brain-Like Intelligence, Shanghai Fourth People’s Hospital, School of Medicine, Tongji University, Shanghai, China

**Keywords:** p62, primary age-related tauopathy, hippocampus, Braak NFT stage, Alzheimier’s disease

## Abstract

The present study analyzed the distribution pattern of p62 immunoreactivity in brains of primary age-related tauopathy (PART) and Braak NFT matched pre-AD and Alzheimer’s disease (AD) patients using immunohistochemistry in combination with semi-quantitative evaluation. In PART and AD brains, p62 was found positive in seven regions, including the neocortex, thalamus, basal ganglia, hippocampus, brainstem, cerebellar dentate nucleus, and the cervical spinal cord. There was a positive correlation between the Braak NFT stage and the distribution of p62 expression. Six stages of expression of p62 were proposed from the present study. Expression of p62 in the hippocampus of PART and AD was classified stage I, the brainstem stage II, the thalamus stage I _I _I, the basal ganglia stage IV, the neocortex stage V, the cerebellum and the cervical spinal cord stage VI. The hippocampus was the site initially affected by p62, especially the CA1 and the subiculum. They might be the earliest accumulation site of p62.

## Introduction

P62, also named sequestosome 1 (SQSTM1), is a selective cargo receptor for autophagy to degrade misfolded proteins (Du et al., 2009). A previous study showed that p62 was associated with neurodegenerative diseases, and one of the underlying mechanisms is p62 induced alteration in autophagy (Liu et al., 2017). p62 induced autophagy failure significantly accelerates aggregation of misfolded proteins. Furthermore, accumulation of misfolded proteins leads to aberrant p62 expression (Liu et al., 2017). Screening with p62 immunohistochemistry has the potential to reveal other types of aggregates that have evaded detection by other stains (Kuusisto et al., 2008). p62-immunoreactivity (IR) is present at a very early stage of inclusion formation. Since it exhibits a high staining intensity of the inclusions with optimal contrast toward the background, it permits reliable evaluation of pathologic lesions (Salminen et al., 2012).

Primary age-related tauopathy (PART) is recently defined by the presence of Alzheimer’s disease (AD)-like neurofibrillary changes without, or with few Aβ plaques. PART can be designated as “Definite” or “Possible” depending on the presence of Aβ plaques. The working classification for definite PART is based on the presence of neurofibrillary tangles (NFT) along with Braak stage ≤ IV and Thal Aβ Phase as 0 (Crary et al., 2014). Based on the neuropathological diagnostic criteria, we found that PART is common in the Chinese population as observed from specimens in our newly established brain bank. It is still unclear whether PART is an early form of AD or a distinct tauopathy different from AD (Duyckaerts et al., 2015; Jellinger et al., 2015). Here, we described the anatomic distribution of p62-IR in PART and AD subjects using immunohistochemistry as a screening stain for diagnostic practice.

### Materials

A total of 18 definite PART and 11 AD cases from the Chinese Brain Bank of School of Medicine, Zhejiang University were examined. All subjects fulfilled the following inclusion criteria: age at death ≥ 50 years, and postmortem delay less than 24 h. The diagnosis of PART was confirmed by a neuropathological investigation with NFT Braak stage ≤ IV and Thal Aβ Phase as 0. AD was confirmed by Braak NFT stage ≥ IV and CERAD plaque density C and pre-AD by Braak NFT stage I _I _I and CERAD plaque density C or B. When used in the NIA-AA diagnostic criteria for AD, the CERAD plaque densities scores are: none = 0, sparse = A, moderate = B, frequent = C (Mirra et al., 1991).

### Immunohistochemistry

Immunohistochemistry of tissues about 605 pieces in total was performed on paraformaldehyde (PFA)-fixed, paraffin-embedded tissue from all autopsy cases. Small blocks of brain were dissected at autopsy and fixed in 4% PFA in 0.1 M phosphate buffer (pH 7.4) for 2 days. Following cryoprotection in 15% sucrose in 0.01 M phosphate-buffered saline (PBS, pH 7.4), blocks were cut into 3 μm thick sections using a microtome and mounted onto slides. Sections were incubated with 3% H_2_O_2_ for 10 min to eliminate endogenous peroxidase activity in the tissue. Prior to immunostaining, sections underwent microwave antigen retrieval for 15 min in the citrate buffer (pH 6.0). After washing with PBS containing 0.3% Triton X-100 (Tx-PBS) for 30 min, sections were blocked with 10% normal serum from the same origin of the secondary antibody, and then incubated with the primary antibody (mouse anti-p62, Biosciences, 610833, 1:100) for 24 h at 4°C. Following incubation with the appropriate secondary antibody, labeling was detected using the avidin–biotinylated HRP complex (ABC) system (Vector Laboratories, Burlingame, CA, United States). The peroxidase reaction was carried out using a developer solution containing 0.4 mg/ml DAB and 0.0006% hydrogen peroxide dissolved in TBS. For the negative control, the primary antibodies were omitted and all other steps carried out as described above. After the staining procedures, sections dehydrated and cleared before being coverslipped with DPX mounting medium. Expression of p62 pathology was semi-quantitatively scored based on a four-point grading scale (-/0, none;+/1, mild; ++/2, moderate;+++/3, severe). Of note, all cases were initially checked using the routine protocol for the Braak staging system (Braak et al., 2006). All images were collected with Virtual Slide System 120 microscope in 10× magnification and analyzed with CellSens Dimension software.

### Ethics Approval and Consent to Participate

Use of tissue for this study was approved by the Medical Ethics Committee of School of Medicine, Zhejiang University. Written consent was obtained with all participants.

### Statistical Methods

All sections were scanned by Virtual Slide System 120 microscope in 10× magnification and analyzed with CellSens Dimension software. IBM SPSS version statistics software (Armonk, NY, United States) was used for statistical analysis, and the standard error of the means was used when applicable. Correlation between the studied variables was assessed using the Spearman correlation analysis. Mann–Whitney U (two groups) test and Kruskal–Wallis H test (three or more groups) were implemented to test the statistical difference between the studied groups. To calculate the quantitative p62 score in hippocampus, three square (100 μm × 100 μm) with same length were selected in each area according to the organizational structures, and the number of p62 positive cells were counted as p62 score.

## Results

### 1. Expression Pattern of p62 in Primary Age-Related Tauopathy

**FIGURE 1:**
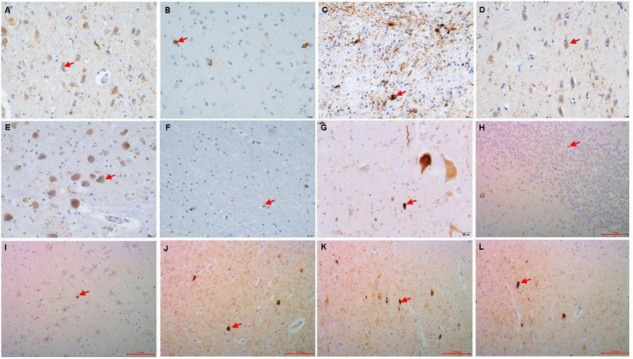
Immunohistochemistry of the p62 + in PART brain area. **(A)** Gyrus pre/postcentralis, **(B)** Hypothalamus, **(C)** Caudatum/Putamen, **(D)** Amygdala, **(E)** Colliculus inferior/pons, **(F)** Cerebellum, **(G)** Spinal cord cervical, **(H)** Granule cell layer of hippocampus dentate gyrus (DG), **(I)** CA4, **(J)** CA1, **(K)** Subiculum (Sub), **(L)** Entorhinal Cortex(EC). Arrow point positive cell. Scale bars, **(A–G)** 20 μm, **(H–L)** 100 μm.

Expression of p62 in PART was assessed in various brain regions (Figure 1), including the neocortex, thalamus, basal ganglia, hippocampus, brainstem, cerebellar dentate nucleus, and the cervical spinal cord. The neocortex includes seven parts: the central anterior gyrus, central posterior gyrus, temporal pole, frontal lobe, cingulate gyrus, the parietal and the occipital poles; the thalamus includes the hypothalamus and subthalamus; the basal ganglia includes the caudate nucleus, putamen, and the globus pallidus; the hippocampus includes the anterior hippocampus, middle hippocampus, and the posterior hippocampus; the brainstem includes the substantia nigra, colliculus inferior/pons (CIP), locus coeruleus/pons (LCP), and the medulla oblongata.

**FIGURE 2:**
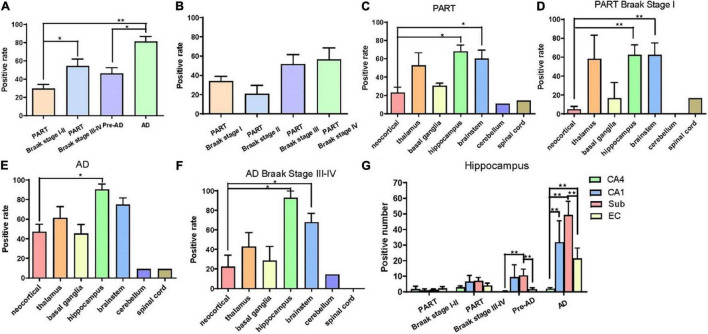
**(A)** Quantification of the positive rate of p62 cell in PART and AD. Values represent mean ± SEM (*n* = 9–16 subjects; **p < 0.01; Kruskal–Wallis H test). **(B)** Quantification of the positive rate of p62 + cell in PART. Values represent mean ± SEM (*n* = 5–9 subjects; Kruskal–Wallis H test). **(C)** Quantification of the positive rate of p62 + cell in PART in brain areas. Values represent mean ± SEM (*n* = 0–9; *p < 0.05; Kruskal–Wallis H test). **(D)** Quantification of the positive rate of p62 + cell in PART in brain areas. Values represent mean ± SEM (*n* = 0–9; **p < 0.01; Kruskal-Wallis H test). **(E)** Quantification of the positive rate of p62 + cell in AD in brain areas. Values represent mean ± SEM (*n* = 0–9; *p < 0.05; Kruskal–Wallis H test). **(F)** Quantification of the positive rate of p62 + cell in AD braak stage III-IV in brain areas. Values represent mean ± SEM (*n* = 8–18; *p < 0.05; Kruskal–Wallis H test). **(G)** Quantification of the positive rate of p62 + cell in hippocampus. Values represent mean ± SEM (*n* = 8–18; **p < 0.01; Mann–Whitney U test).

Overall, the positive rate of p62 increased with the increase of Braak NFT stage (Figure 2A) (*p < 0.05; ^**^p < 0.01). Among the four groups, the positive rate of p62 was low in PART with Braak NFT stage I/II, moderate in PART with Braak NFT stage I _I _I/IV, which was close to that of pre-AD, and high in AD with Braak stage V/VI. There were statistical differences between PART with Braak NFT stage I/II and PART Braak NFT stage I _I _I/IV as well as between PART with Braak NFT stage I/II and AD, and between pre-AD and AD. Among the four groups of PART, positive expression of p62 increased with the increase of Braak NFT stage, but there was no statistical difference between these PART groups (Figure 2B).

Three regions with the highest expression of p62 in PART were the hippocampus, brainstem, and the thalamus, and there were significant differences (*p* < 0.05) between the neocortex and the hippocampus as well as between the brainstem and the neocortex (Figure 2C). Expression of p62 in PART cases with NFT Braak stage I was highest in the hippocampus and the brainstem, and there was a statistical difference (*p* < 0.01) between these two regions and the neocortex, respectively. P62 was not detectable in the cerebellum in PART cases with NFT Braak stage I (Figure 2D).

**TABLE 1:** p62-positive structures in primary age-related tauopathy (PART).

PART	Age	Sex	Neocortical							Thalamus		Basal ganglia		Hippocampus				Brainstem				Ce	SC
			GPP	T	F	GC	LPI	P	O	H	TS	CP	PPI	A	HA	HM	HP	SN	CIP	LCP	MO		
I	60	M	–	+	–	–	–	–	–	+	–	–	+	–	–	–	+	–	+	+	–	–	–
	74	F	–	–	–	–	–	–	–	+	–	–	–	+	+	+	+	+	+	–	–	–	–
	76	M	–	–	–	–	–	–	–	+	+	–	+	–	+	–	+	+	+	+	+	–	+
	84	F	–	–	–	–	–	–	–	–	–	–	–	–	–	+	–	+	–	–	–	–	–
	91	M	–	–	–	–	–	–	+	+	–	–	–	–	+	+	+	+	+	–	+	–	–
	95	M	–	–	–	–	–	–	–	+	+	–	–	+	+	+	+	+	+	–	+	–	–
II	65	M	–	+	–	–	–	+	–	+	–	+	–	–	+	+	+	–	+	–	–	–	–
	77	M	–	–	–	–	–	–	–	–	–	–	–	–	+	–	–	+?	+	+	–	–	–
	83	M	–	–	–	–	–	–	–	–	–	–	–	–	+	–	–	–	+	–	–	–	N
III	80	F	–	+	–	–	–	+	–	+	–	–	–	+	+	–	+	+	+		–	–	–
	80	M	–	+	–	+	+	+	–	+	+	+	+	+	+	+	+	+	+	+	+	–	+
	81	F	+	–	–	–	–	–	–	+	–	–	+	–	–	–	+	+	+	+	+	–	–
	83	F	+	+	–	+	–	–	–	–	–	–	–	+	+	+	+	+	+	–	+	–	–
IV	60	F	+	+	+	+	+	+	+	+	+	+	+	–	+	+	+	+	+	+	+	+	
	74	M	–	+	+	+	–	–	+	+	+	+	+	+	+	+	+	+	–	+	–	–	–
	78	M	–	+		–	–	–	–	–	–	–	–	+	–	+	+	–	+	+	–	–	
	79	M	–	+	–	–	–	+	–	–	+	–	–	+	–	+	+	–	–	–	+	–	
	98	M	–	+		–	–	–	–	+	+	+	–	+	+	+	+	+	+	–	–	–	–

*GPP, gyrus pre/postcentralis; T, Temporal pole; F, frontal lobe; GC, gyrus cinguli; LPI, lobular parietal inferior; P, parietal; O, occipital lobe; H, hypothalamus; TS, thalamus/subthalamus; CP, caudatum/putamen; PPI, putamen/pallidum/Insular; AE, amygdala/entorhinal cort; HA, hippocampus anterior; HM, hippocampus middle; HP, hippocampus post; SN, substantia nigra; CIP, colliculus inferior/pons; LCP, Loc. coeruleus/pons; MO, medulla oblongata; Ce, cerebellum; SC, spinal cord cervical.*

### 2. Expression Pattern of p62 in Alzheimer’s Disease

The distribution pattern of p62-IR in the same brain regions of 11 AD cases with different NFT Braak stages was examined and compared with that of PART cases (Table 2). The positive rate of p62 was higher in AD than in PART. The highest expression was found in the hippocampus, and there was a statistical difference between the hippocampus and the neocortex (Figure 2E) (p < 0.05). When pre-AD (Braak NFT stage I _I _I/IV) was analyzed separately, the highest expression was found in the hippocampus, followed by the brainstem. There were significant differences(p < 0.05) between these two regions and the neocortex, respectively (Figure 2F). The positive rates of p62 in PART and AD were ranked in the order of the hippocampus, brain stem, thalamus, basal ganglia, neocortex, cerebellum, and the spinal cord (Figures 2C,E).

**TABLE 2:** p62-positive structures in Alzheimer’s disease (AD).

PART	Age	Sex	Neocortical							Thalamus		Basal ganglia		Hippocampus				Brainstem				Ce	SC
			GPP	T	F	GC	LPI	P	O	H	TS	CP	PPI	AE	HA	HM	HL	SN	CIP	LCP	MO		
III	69	F	–	–	–	–	–	–	–	–	–	–	–	+	+	+	+	+	–	+	+	–	–
	73	M	–	+	+	++	–	–	–	+	+	+	+	+	+	+	+	+	++	+	++	–	
	79	F	–	+	–	–	–	–	–	–	+	–	+	+	+	+	+	–	+	+	–	+	–
	93	F	+	+	–	+	–	–	–	–	–	–	–	+	++	+	–	+	–	–	–	–	–
IV	78	M	–	+	–	–	–	–	–	+	–	–	–	+	+	+	–	–	+	+	+	–	–
	81	F	–	+	+	++	–	–	–	+	–	–	+	++	+++	+	++	–	+	+	+	–	–
	83	M	–	+	–	–	–	–	–	+	–	–	–	+++	+	+	+	–	+	+	+	–	–
V	98	F	–	–	+	+	++	++	+	+	+	–	–	+++	+++	+	++	–	+	+	+	–	–
VI	79	M	+	+	+++	+++	++	+++	+++	++	++	+	+++	++	+++	+	++	+	+	–	+	–	–
	85	M	–	+	+	++	++	+	+++	+++		+++	+++	+	+++	+		+	+	+	+	–	–
	87	F	+	+	+++	+++		+++	+	+	++	+	+++	+++	+++	+	+++	+	++	+	+	–	–

*GPP, gyrus pre/postcentralis; T, temporal pole; F, frontal lobe; GC, gyrus cinguli; LPI, lobular parietal inferior; P, parietal; O, occipital lobe; H, hypothalamus; TS, thalamus/subthalamus; CP, caudatum/Putamen; PPI, putamen/pallidum/insular; AE, amygdala/entorhinal cort; HA, hippocampus anterior; HM, hippocampus middle; HP, hippocampus post; SN, substantia nigra; CIP, colliculus inferior/pons; LCP, Loc. coeruleus/pons; MO, medulla oblongata; Ce, cerebellum; SC, spinal cord cervical.*

### 3. Expression Pattern of p62 in the Hippocampus

Five regions of the hippocampus were checked, including the CA1, CA4, dentate gyrus, subiculum, and the entorhinal cortex, and semi-quantitative analysis was performed. The number of p62 positive cases in the AD group was significantly larger than in the pre-AD group, PART with Braak NFT stage I/II and PART with Braak NFT stage I _I _I/IV groups (Figure 2G). In the hippocampus of AD cases, the highest expression of p62 was found in the subiculum, followed by the CA1, the entorhinal cortex, and the CA4. p62 was rarely observed in the dentate gyrus. There were statistical differences between CA4 and the other three regions, respectively (*p* < 0.01). In pre-AD, the highest expression of p62 was found in the subiculum, followed by the CA1, the entorhinal cortex, and the CA4. There were significant differences between the subiculum and the entorhinal cortex as well as between the subiculum and the CA4 (*p* < 0.01) (Figure 2G).

## Discussion

It remains to be determined whether p62 plays a role in PART. When anatomical regions were taken into consideration, we found that expression of p62 increased with the increase in Braak stages in both PART and AD. We also showed that p62 expression was highest in the hippocampus and the brainstem, especially in the subiculum and the CA1 of the hippocampus, and detected early in these regions. p62 expression was positive in the neocortex at later stages. These data provide the first direct evidence showing p62 expression in different brain regions in PART.

**FIGURE 3:**
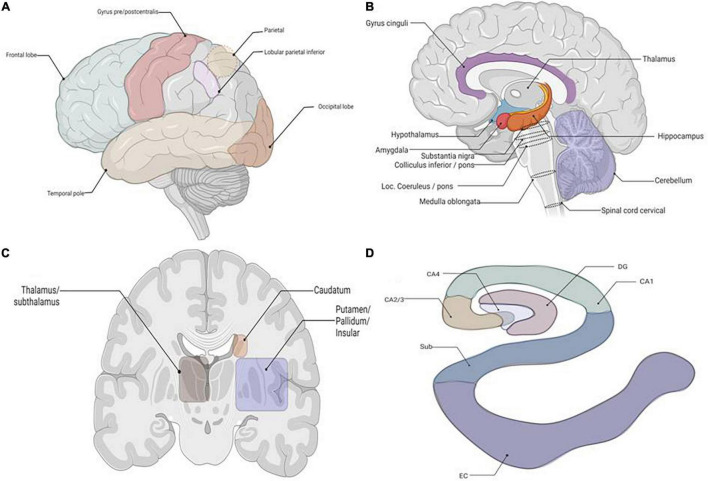
The schematic about the location of the tissues required for p62 staining. **(A)** Lateral view of brain, all lobes. **(B)** Sagittal cut of brain. **(C)** Coronal cut at basal ganglia. **(D)** Coronal cut of brain at hippocampus. Granule cell layer of dentate gyrus (DG), Subiculum (Sub), Entorhinal Cortex (EC).

Expression of p62 was detected in multiple brain regions in PART, pre-AD, and AD brains with the highest expression in the hippocampus, followed by the brainstem and the thalamus (Figure 3). We lack the p62 expression data in the normal brain. This is our limitation. Therefore, it is difficult to infer that p62 expression of AD or PART is higher than that of normal controls. Here we focus on p62 expression of AD and PART. There was a positive correlation between expression of p62 and Braak stages in both PART and AD. Within the hippocampus, the subiculum had the highest level of p62, followed by the CA1 and other regions. In our previous research, we demonstrated regional patterns of selective vulnerability as distinguishing features of PART and AD in functionally relevant structures of the hippocampus. In AD cases, tau pathology was high in both CA1 and subiculum, followed by CA2/3, entorhinal cortex (EC), CA4, and dentate gyrus (DG). In PART, the severity of tau pathology in CA1 and subiculum was high, followed by EC, CA2/3, CA4, and DG (Zhang et al., 2020). These suggest that the subiculum of the hippocampus might be the earliest brain region that presents with AD pathology.

**TABLE 3:** The quantitative p62 score in hippocampus.

Braak stage		Age	Sex	CA4	CA1	DG	Sub	EC
PART	I	60	M	0	0	0	0	1
		74	F	9	4	0	3	2
		84	F	1	2	0	2	2
		91	M	0	0	0	1	0
	II	65	M	1	0	0	0	10
		77	M	0	1	0	2	1
		95	M	0	0	0	1	7
	III	76	M	6	41	0	13	1
		80	M	8	1	0	5	0
		80	F	1	8	0	23	9
	IV	60	F	1	2	0	4	2
		74	M	3	2	0	1	1
		78	M	0	2	0	3	0
		79	M	7	4	1	10	2
AD	III	69	F	0	1	0	6	0
		73	M	1	2	0	7	4
		93	F	1	0	0	12	5
	IV	78	M	0	3	0	5	0
		81	F	0	2	0	3	1
		83	M	1	49	0	30	0
	V	98	F	3	78	0	74	27
	VI	79	M	1	47	0	60	45
		85	M	0	4	0	46	14
		87	F	4	22	3	22	13

*DG, dentate gyrus; Sub, subiculum; EC, entorhinal cortex.*

Six stages of p62 expression are proposed from the present study. At stage I, p62 immunoreactive inclusions are present in neurons of the hippocampus. At stage II, p62 expression expands to involve neurons of the brainstem. At stage I _I _I, the pathological inclusions spread to the thalamus. At stage IV, the basal ganglia show p62 expression. At stage V, the neocortical neurons show p62 positive inclusions. At Stage VI, the cerebellum shows p62 expression. These stages might represent disease progression in a sense. It has a pathological meaning in the clinical practice.

The multifunctional protein p62 is associated with neuropathological inclusions in various neurodegenerative disorders, including frontotemporal lobar degeneration (FTLD), amyotrophic lateral sclerosis and AD (Ma et al., 2019; Deng et al., 2020). Strong evidence has shown that p62 immunoreactivity is associated with neurofibrillary tangles formation (Kuusisto et al., 2002). In general, p62 protein is not a specific marker. Immunohistochemistry against p62 shows potential to facilitate neuropathological diagnosis by displaying a general pattern of stained inclusions. The use of p62 immunodetection was recommended as a sensitive diagnostic screening test for assessment of degenerative dementias, including FTLD-U (Pikkarainen et al., 2008).

Previous studies have shown that p62 participates in the formation of NFT in AD. p62 could be an early marker for neurodegenerative changes in PART, AD, and other neurodegenerative diseases (Piras et al., 2016). In the present study, we found that p62 was positive at the early stage of PART with Braak stage I/II and its expression was positively correlated with the NFT Braak stage. This supports that p62 might be involved in the aggregation of NFT at the early stage of AD (Zhang et al., 2020). In this study, p62 first appeared in the hippocampus. This is different from results demonstrating that LC is the earliest site of NFT formation (Braak et al., 2011; Attems et al., 2012).

## Data Availability Statement

The raw data supporting the conclusions of this article will be made available by the authors, without undue reservation.

## Ethics Statement

The studies involving human participants were reviewed and approved by family members of brain donors. This research was approved by the Medical Ethics Committee of Zhejiang University School of Medicine. The patients/participants provided their written informed consent to participate in this study.

## Author Contributions

XW, HLu, JW, and BS prepared the tissue for immunohistochemistry and performed statistical analysis. KZ and LZ contributed to the statistical assessment and data processing. KZ designed the study, analyzed the results, and wrote the first version of the manuscript which was circulated among all the contributors for comments and suggestions. HLi contributed to the final version of the manuscript. All authors contributed to the article and approved the submitted version.

## Conflict of Interest

The authors declare that the research was conducted in the absence of any commercial or financial relationships that could be construed as a potential conflict of interest.

## Publisher’s Note

All claims expressed in this article are solely those of the authors and do not necessarily represent those of their affiliated organizations, or those of the publisher, the editors and the reviewers. Any product that may be evaluated in this article, or claim that may be made by its manufacturer, is not guaranteed or endorsed by the publisher.
